# Molecular and clinicopathologic features of gliomas harboring *NTRK* fusions

**DOI:** 10.1186/s40478-020-00980-z

**Published:** 2020-07-14

**Authors:** Matthew Torre, Varshini Vasudevaraja, Jonathan Serrano, Michael DeLorenzo, Seth Malinowski, Anne-Florence Blandin, Melanie Pages, Azra H. Ligon, Fei Dong, David M. Meredith, MacLean P. Nasrallah, Craig Horbinski, Sonika Dahiya, Keith L. Ligon, Mariarita Santi, Shakti H. Ramkissoon, Mariella G. Filbin, Matija Snuderl, Sanda Alexandrescu

**Affiliations:** 1 Department of Pathology, Brigham and Women’s Hospital and Harvard Medical School, 75 Francis Street, Boston, MA 02115 USA; 2 Department of Pathology, Boston Children’s Hospital and Harvard Medical School, 300 Longwood Ave, Bader Building, Boston, MA 02115 USA; 3 Department of Pathology, NYU Langone Health, 550 First Avenue, New York, NY 10016 USA; 4 Department of Oncologic Pathology, Dana-Farber Cancer Institute, 450 Brookline Avenue, Boston, MA 02115 USA; 5 Department of Neuropathology, GHU Paris Sainte-Anne Hospital, 1 Rue Cabanis, 75014 Paris, France; 6 Center for Advanced Molecular Diagnostics, Brigham and Women’s Hospital and Harvard Medical School, 75 Francis Street, Boston, MA 02115 USA; 7 Department of Pathology and Laboratory Medicine, Perelman School of Medicine, University of Pennsylvania, 3400 Spruce Street 34th St, Philadelphia, PA 19104 USA; 8 Department of Neurological Surgery, Northwestern University, Chicago, IL USA; 9 Department of Pathology, Northwestern University, 303 East Chicago Avenue, Chicago, IL 60611 USA; 10 Division of Neuropathology, Department of Pathology and Immunology, Washington University School of Medicine, 660 South Euclid Avenue, Campus Box 8118, St. Louis, MO 63110 USA; 11 Department of Pathology and Laboratory Medicine, Children’s Hospital of Philadelphia, Philadelphia, PA USA; 12 Foundation Medicine, 7010 Kit Creek Road, Morrisville, NC 27560 USA; 13 Wake Forest Comprehensive Cancer Center and Department of Pathology, Wake Forest School of Medicine, Winston-Salem, 27157 NC USA; 14 Department of Pediatric Oncology, Dana Farber Cancer Institute, Harvard Medical School, 450 Brookline Avenue, Boston, MA 02215 USA

**Keywords:** *NTRK*, Glioma, NGS sequencing, Methylation

## Abstract

Fusions involving neurotrophic tyrosine receptor kinase (*NTRK*) genes are detected in ≤2% of gliomas and can promote gliomagenesis. The remarkable therapeutic efficacy of TRK inhibitors, which are among the first Food and Drug Administration-approved targeted therapies for *NTRK*-fused gliomas, has generated significant clinical interest in characterizing these tumors. In this multi-institutional retrospective study of 42 gliomas with *NTRK* fusions, next generation DNA sequencing (*n* = 41), next generation RNA sequencing (*n* = 1), RNA-sequencing fusion panel (*n* = 16), methylation profile analysis (*n* = 18), and histologic evaluation (*n* = 42) were performed. All infantile *NTRK*-fused gliomas (*n* = 7) had high-grade histology and, with one exception, no other significant genetic alterations. Pediatric *NTRK*-fused gliomas (*n* = 13) typically involved *NTRK2*, ranged from low- to high-histologic grade, and demonstrated histologic overlap with desmoplastic infantile ganglioglioma, pilocytic astrocytoma, ganglioglioma, and glioblastoma, among other entities, but they rarely matched with high confidence to known methylation class families or with each other; alterations involving *ATRX*, *PTEN*, and *CDKN2A/2B* were present in a subset of cases. Adult *NTRK*-fused gliomas (*n* = 22) typically involved *NTRK1* and had predominantly high-grade histology; genetic alterations involving *IDH1*, *ATRX*, *TP53*, *PTEN*, *TERT* promoter, *RB1*, *CDKN2A/2B*, *NF1*, and polysomy 7 were common. Unsupervised principal component analysis of methylation profiles demonstrated no obvious grouping by histologic grade, *NTRK* gene involved, or age group. KEGG pathway analysis detected methylation differences in genes involved in PI3K/AKT, MAPK, and other pathways. In summary, the study highlights the clinical, histologic, and molecular heterogeneity of *NTRK*-fused gliomas, particularly when stratified by age group.

## Introduction

The tropomyosin receptor kinase (TRK) family of tyrosine receptor kinases is comprised of TRKA, TRKB, and TRKC, which are encoded by neurotrophic tyrosine receptor kinase (NTRK) genes *NTRK1*, *NTRK2*, and *NTRK3*, respectively. The three TRK proteins are structurally similar, with an extracellular region containing leucine-rich repeats, cysteine-rich clusters, and immunoglobulin-like domains, a transmembrane region, and an intracellular region including a tyrosine kinase domain [2]. Binding of neurotrophin ligands to the extracellular region triggers TRK dimerization and transphosphorylation of tyrosine residues within the activation loop of the kinase domain, which ultimately results in the upregulating of multiple pathways including mitogen-activated protein kinase (MAPK), phosphatidylinositol-3-kinase / protein kinase B (PI3K/AKT), and phospholipase C-γ (PLC-γ) signaling cascades [27]. TRK receptors are highly expressed in neural tissue, where they have a physiologic role in neuronal survival, development, proliferation, and synaptic plasticity, as well as memory and cognition [2].

Fusions involving the *NTRK* genes can be oncogenic drivers and typically involve the 5′ end of the fusion partner and the 3′ end of *NTRK* preserving the tyrosine kinase domain. Reported gene fusion partners are numerous and in many cases contain structural motifs such as coiled-coil domains and zinc finger domains that promote dimerization [10]. Thus, oncogenic *NTRK* fusions can result in aberrant ligand-independent TRK receptor dimerization and constitutive activation of TRK signaling pathways [3], leading to upregulated proliferation and resistance to apoptosis. *NTRK* fusions in which the fusion partners lack dimerization domains might alternatively promote tumorigenesis through loss of extracellular TRK regulatory domains [5].

The estimated prevalence of *NTRK*-fusions across all tumors is less than 1% [34, 38]. However, for certain tumors such as congenital infantile fibrosarcoma, mammary analogue secretory carcinoma, and secretory breast carcinoma, *NTRK* fusions occur in greater than 90% of cases and are essentially pathognomonic for those entities [48]. *NTRK* fusions occur in lower frequencies in a wide range of other neoplasms, including colorectal carcinoma, lung carcinoma, and papillary thyroid carcinoma, among others [48]. Approximately 0.55 to 2% of all gliomas/neuroepithelial tumors contain *NTRK* fusions [18, 21, 34, 38, 43, 53], though the incidence may be up to 5.3% in pediatric high grade gliomas (HGG) [34], 4% of diffuse intrinsic pontine gliomas (DIPG), and 40% of non-brainstem HGG in patients younger than 3-years-old [52].

Clinical interest in *NTRK*-fused tumors has increased substantially due to the efficacy of Food and Drug Administration (FDA) approved TRK inhibitor therapies [4, 13, 15–17, 23, 30]. The aim of the current study is to provide insights into the clinicopathologic and molecular features of gliomas with *NTRK* fusions.

## Materials and methods

### Cohort

The surgical, consultation, and molecular pathology archives of Brigham and Women’s Hospital (BWH) (Boston, MA), Boston Children’s Hospital (BCH) (Boston, MA), Children’s Hospital of Philadelphia (CHOP) (Philadelphia, PA), Washington University School of Medicine (WashU) (St Louis, MO), Northwestern University (NWU) (Chicago, IL), and Foundation Medicine (FM) (Morrisville, NC) were reviewed for gliomas with *NTRK* rearrangements. In this retrospective multi-institutional study, a total of 42 cases were identified, composed of 8 cases from BWH, 7 cases from BCH, 5 cases from CHOP, 1 case from WashU, 1 case from NWU, and 20 cases from FM. The study contains 2 cases (cases 7 and 15) that have been previously reported in the literature [31, 46]. The study was conducted under BCH IRB protocol IRB-CR00027359–1 and DFCI protocol 10–417. The cases were grouped in infantile (age less than 1 year), pediatric (age ranging from 1 year to 18 years), and adult (age over 18 years). Available routine hematoxylin and eosin stained sections and immunohistochemical stains prepared from formalin-fixed, paraffin-embedded (FFPE) tissue from the 42 identified cases underwent review by neuropathologists (SA, MT, SHR, MSa, CH), with 22 of the cases undergoing central review by SA; all tumors with material for methylation were centrally-reviewed. In general, there was agreement with the initial clinical diagnosis, and a specific World Health Organization (WHO) diagnosis was sought whenever the histology allowed it. A complete set of slides was not available for the 20 cases from FM; however, these were all reviewed by one neuropathologist (SHR). A subset of the pediatric tumors had concerning histology, with occasional mitoses and pleomorphism, but, overall, these features did not reach the threshold for WHO histologic grade 3. The histologic diagnoses in cases that posed this challenge were: glioma with anaplastic features (4 cases) and anaplastic pilocytic astrocytoma (APA) (1 case). Therefore, a specific histologic grade could not be assigned for these tumors and they are referred to as having “uncertain WHO histologic grade” in the manuscript. Patient information was abstracted from the electronic medical records or from the clinical information provided on the pathology report.

### Figures

The Oncoprint figure was created using R 3.6.0, RStudio 1.2.1335, and the Oncoprint function of the C*omplexHeatmap* 2.2.0 package. The Circos plot was generated using the online Circos Table Viewer (http://mkweb.bcgsc.ca/tableviewer). All other figures were created using GraphPad Prism (v.8) software.

### Next generation sequencing (NGS)

*NTRK* rearrangements were detected by either DNA-based next generation sequencing (NGS) or RNA-based fusion panel performed at the time of clinical diagnosis. Given that this is a retrospective multi-institutional study, a limitation is that the NGS panels utilized are institution-specific (albeit similar in coverage of genes of interest and scope).

The NGS platforms used included the BWH hybrid capture sequencing assay (Oncopanel) (*n* = 12), Foundation Medicine hybrid capture sequencing assay (*n* = 22, including 1 case from WashU and 1 case from NWU), CHOP Comprehensive Solid Tumor Panel (*n* = 6), GlioSeq NGS panel (n = 1), Caris Life Sciences NGS panel (n = 1), and Integragen Genomics (next generation RNA sequencing) (n = 1) (https://www.integragen-genomics.com/bioinformatics-and-bioanalysis/mercury). In addition, RNA-based fusion testing was performed on 16 cases either as standalone targeted RNA-based anchored multiplex PCR (Archer FusionPlex) [56] or as part of a multi-assay panel (e.g. CHOP Comprehensive Solid Tumor Panel, GlioSeq NGS panel, Caris Life Sciences NGS panel).

Oncopanel interrogates the exons of 447 genes and 191 introns across 60 genes, and structural rearrangements are evaluated with BreaKmer analysis as previously described [20]. The Foundation Medicine NGS assay evaluates 324 genes for mutations and copy number alterations, as well as select intronic regions of a subset of genes to detect gene rearrangements. Details about the Foundation Medicine NGS assay can be found at https://www.foundationmedicine.com/genomic-testing/foundation-one-cdx. The CHOP Comprehensive Solid Tumor Panel includes sequencing and copy number analysis of 237 genes as well as targeted RNA-based anchored multiplex PCR using custom probes for over 100 genes, as previously described [49]. Caris Life Sciences performs exome sequencing on 592 genes for mutational analysis, evaluates a proportion of these genes for copy number alterations, and assesses for fusions involving targeted genes with RNA-based anchored multiplex PCR (https://www.carismolecularintelligence.com/profiling-menu/mi-profile-usa-excluding-new-york/). GlioSeq uses amplification-based DNA and RNA sequencing to evaluate for mutations, copy number alterations, and structural rearrangements involving genes relevant to primary central nervous system (CNS) tumors. A list of genes included in the GlioSeq panel can be accessed at https://mgp.upmc.com/Home/Test/GlioSeq_details. Copy number data was determined from DNA-based NGS and methylation profile plots.

### DKFZ CNS tumor classification of *NTRK* gliomas

Genome-wide methylation profiling was performed on DNA extracted from FFPE tissue from 18 cases with available material using the Illumina EPIC Array 850 Bead-Chip (850 k) array to evaluate the DNA methylation status of over 850,000 CpG sites, as described previously [40]. The raw idat files were then analyzed by the Brain Tumor Classifier developed by Capper et al. [7], which is clinically validated at NYU. Each *NTRK* fusion case was compared against the CNS reference tumor cohort (82 methylation classes and 9 control tissues) using the Random Forest Classifier. The classifier generates Methylation classifier scores for each sample along with t-distributed stochastic neighbor embedding (tSNE) dimensionality reduction of queried samples against the reference cohort classes.

### *NTRK* cohort genome-wide methylation profiling and analysis

To analyze the *NTRK* cohort in our study, the raw idats generated from iScan were processed and analyzed using Bioconductor R package *Minfi*. All the Illumina EPIC array probes were normalized using quintile normalization and corrected for background signal. Samples were then checked for their quality using mean detection *p*-values (p-value < 0.05). Unsupervised principal component analysis (PCA) was performed to check for biological variation within the cases. To identify the differentially methylated CpG probes, the samples were grouped based on *NTRK* gene involved, histologic grade, and age. Beta values were generated and probes with FDR cutoff (q < 0.05) were considered the most significantly variable probes. Heatmaps were generated in a semi-supervised manner, showing the hierarchical clustering pattern of the top 10,000 significant differentially methylated genes/probes by *NTRK* gene involved. KEGG pathway analysis with ClusterProfiler [54] was used to identify the signaling pathways enriched in the top 10,000 most variable genes/probes.

The types of molecular assays performed on each case are listed in Supplemental Table 1.

## Results

### Clinical characteristics

**Fig. 1:**
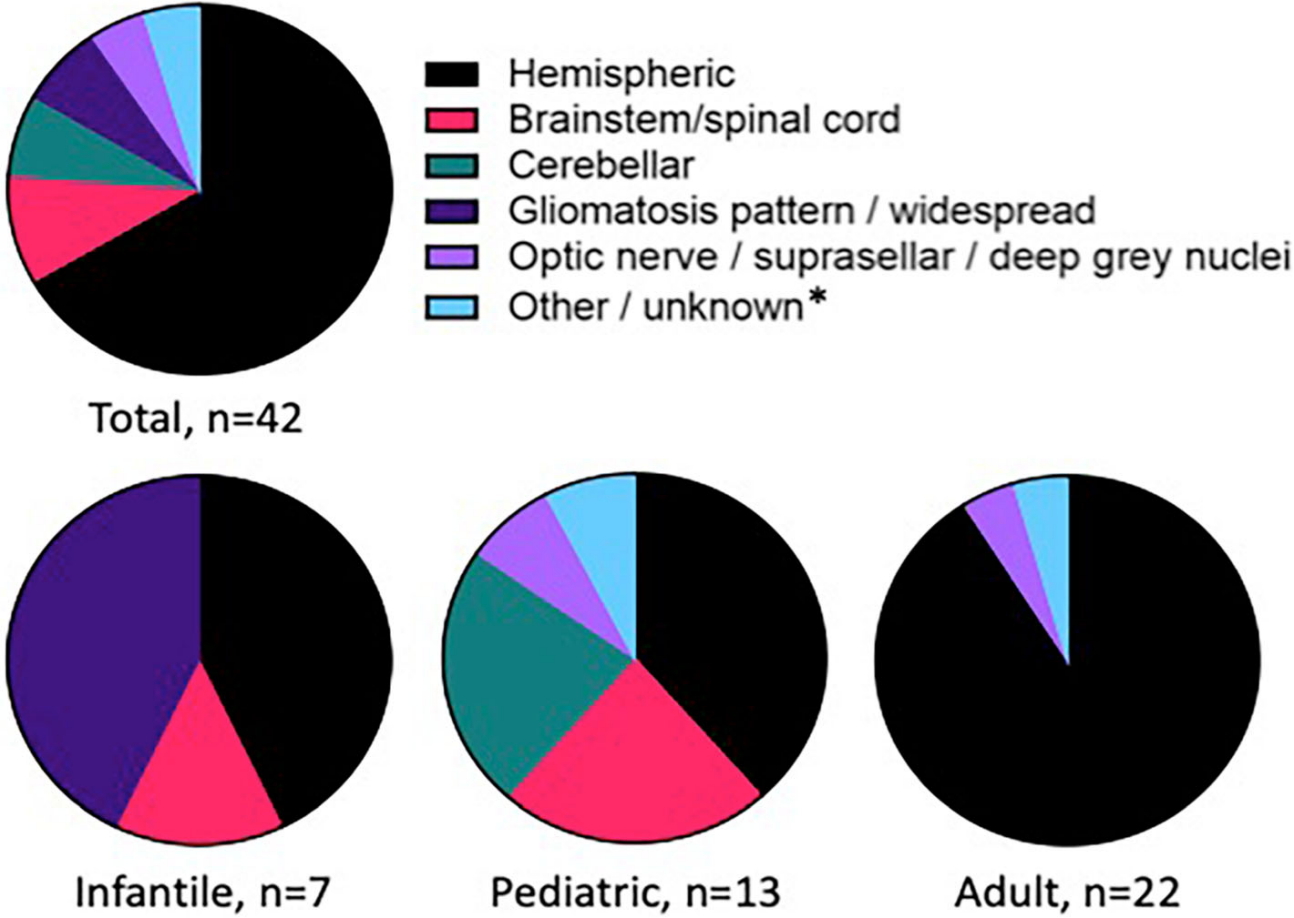
Anatomic distribution of *NTRK*-fused gliomas: *NTRK*-fused gliomas are primarily hemispheric, particularly in infant and adult patients. *NTRK*-fused gliomas in pediatric patients are more diverse in anatomic location. *Includes case involving septum pellicidum

The cohort was comprised of 42 patients (24 males (57.1%), 18 females (42.9%)). The median patient age was 24-years-old (range < 1 to 81 years), with 7 cases arising in infants (age ≤ 1 year), 13 in pediatric patients (age ranging from > 1 to ≤18 years), and 22 in adult patients (age > 18 years). Collectively, most *NTRK*-fused gliomas were hemispheric (66.7%, 28/42), but also involved brainstem/spinal cord (9.5%, 4/42), cerebellum (7.1%, 3/42), optic nerve/suprasellar region/deep grey nuclei (4.8%, 2/42), septum pellucidum (2.8%, 1/42), or had a gliomatosis or widespread pattern (7.1%, 3/42); the location is not known in one case. The distribution of anatomic regions involved varied by age: while the infantile and adult *NTRK*-fused gliomas were typically hemispheric, the anatomic locations of pediatric *NTRK*-fused gliomas were more diverse: 38.5% (5/13) were hemispheric, 23.1% (3/13) were cerebellar, 23.1% (3/13) involved brainstem/spinal cord, 7.7% (1/13) were suprasellar, and 7.7% (1/13) involved the septum pellucidum. The anatomic distribution of *NTRK*-fused gliomas is summarized in Fig. 1.

Survival and progression-free survival data were available in 21 cases (4 infantile, 9 pediatric, and 8 adult patients). For 2 infantile cases, a diagnosis was made at autopsy. Excluding these 2 cases, the median follow-up period after diagnostic procedure was 23 months (range 4–189 months). During this time, 57.9% (11/19) of cases showed tumor recurrence/progression. Cases with recurrence (*n* = 11) were mostly either high histologic grade (WHO grade 3 or 4) (54.5%, 6/11) or of uncertain WHO grade (36.4%, 4/11), and only one case was of low histologic grade (9.1%, 1/11). Both infantile *NTRK*-fused gliomas demonstrated progression/recurrence (100%, 2/2); in the pediatric and adult age groups, tumor progression/recurrence occurred in 55.6% (5/9) and 50.0% (4/8) of cases, respectively. Death occurred in 28.6% (6/21) of cases (2 infantile and 4 adult patients), all with high-grade histology. Patient clinical characteristics are summarized in Table 1.

**Table 1:** Clinicopathologic characteristics

Patient characteristics	Total, n (%)
Total Number of Patients	42 (100%)
Demographics	
Sex	
Male	24 (57.1%)
Female	18 (42.9%)
Age, median (range), years	24 (<1-81)
Infantile (≤1), n (%)	7 (16.7%)
Pediatric (>1 to ≤18), n (%)	13 (31.0%)
Adult (>18), n (%)	22 (52.4%)
Radiology	
Location	
Hemispheric	28 (66.7%)
Brainstem/spinal cord	4 (9.5%)
Cerebellar	3 (7.1%)
Gliomatosis pattern/widespread	3 (7.1%)
Optic nerve/suprasellar/deep grey nuclei	2 (4.8%)
Other/unknown	2 (4.8%)
Survival Data (*n*=21: 4 infantile, 9 pediatric, 8 adult) (cases without any available follow-up data excluded)	
Deaths	6 (28.6% of all cases; all HG)
Infantile	2 (50%)
Pediatric	0 (0%)
Adult	4 (50%)
Tumor Recurrence/Progression	11 (57.9%; 6 HG, 1 LG, 4 of certain WHO grade)^a^
Infantile	2 (100%)^a^
Pediatric	5 (55.6%)
Adult	4 (50%)
Follow-up, median (range), months	23 (4-189)^a^

^a^Excludes 2 infantile tumors diagnosed at autopsy

*HG* High histologic grade, *LG* Low histologic grade

**Fig. 2:**
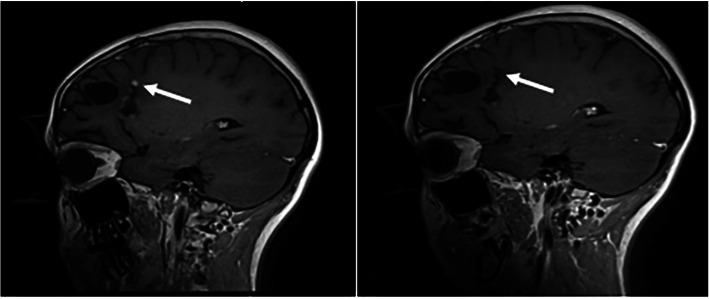
Representative T1 postcontrast MRI images of a pediatric *NTRK*-fused glioma treated with targeted TRK inhibitor therapy. (**a**) Recurrent/residual tumor along the patient’s resection cavity (arrow) demonstrated (**b**) radiologic response to larotrectinib (arrow)

Targeted therapy with larotrectinib was administered in 3 patients: 2 pediatric patients (one of whom showed a decrease in tumor burden (Fig. 2a-b), and one of whom has shown stable disease), and 1 infantile patient whose course of larotrectinib was terminated due to elevated liver function tests.

### *NTRK* fusions

**Fig. 3:**
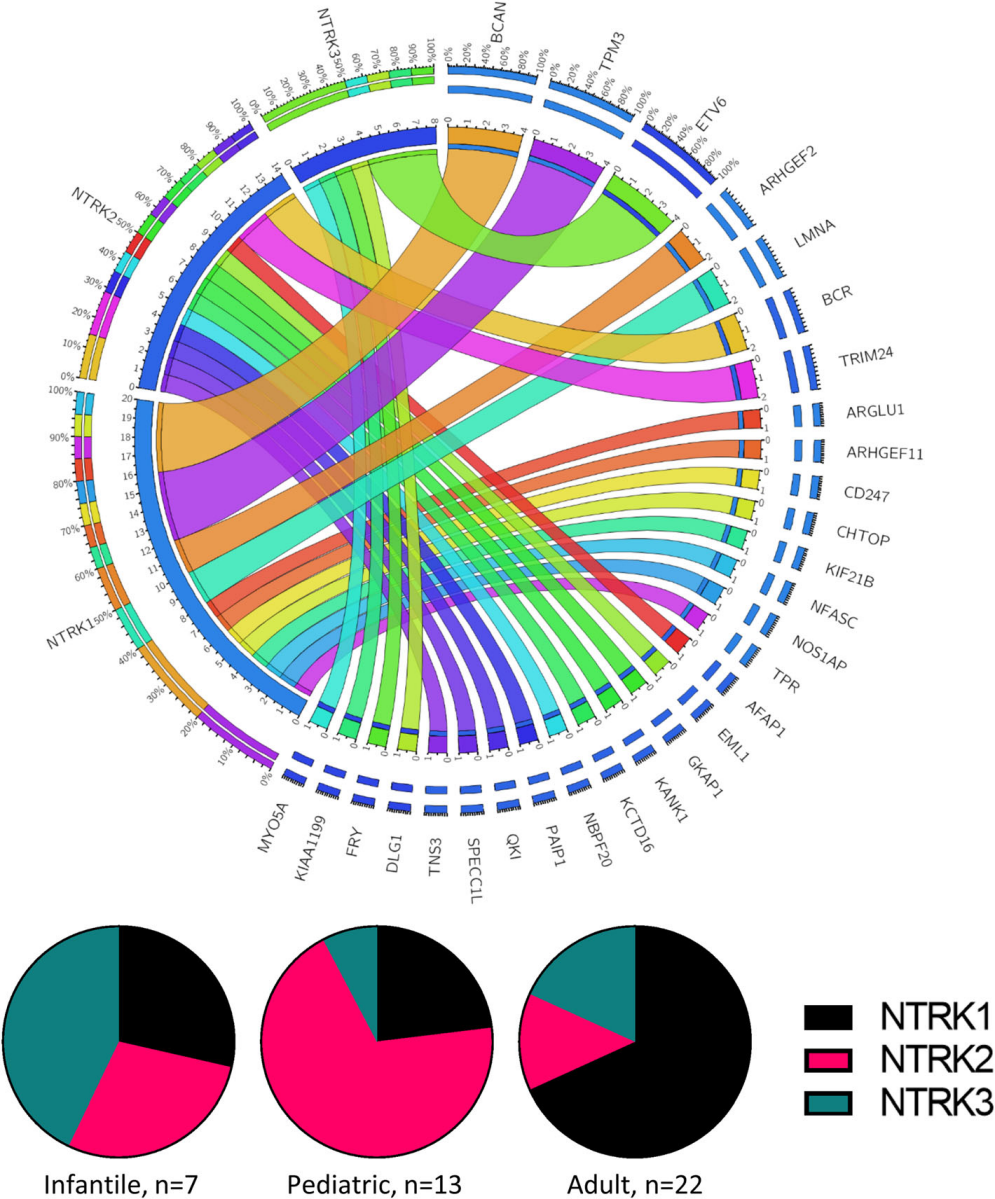
*NTRK* fusions: (Top) Circos plot showing *NTRK* fusions from all 42 cases in the study cohort. There were a total of 29 unique fusion partners, with several recurrent fusions (*BCAN-NTRK1*, *TPM3-NTRK1*, *ETV6-NTRK3*, *ARHGEF2-NTRK1*, *LMNA-NTRK1*, *BCR-NTRK2*, and *TRIM24-NTRK2*). (Bottom): The frequencies of *NTRK* genes involved in the rearrangements varied by age group

*NTRK* rearrangements involved *NTRK1* in 47.6% of cases (20/42), *NTRK2* in 33.3% of cases (14/42), and *NTRK3* in 19.0% (8/42) of cases (Fig. 3). The frequencies of *NTRK* genes involved varied by patient age. All *NTRK* genes were involved in infantile *NTRK*-fused gliomas in approximately equal proportions. In comparison, the majority of pediatric *NTRK*-fused gliomas involved *NTRK2* (69.2%, 9/13). Most adult *NTRK*-fused gliomas involved *NTRK1* (68.2%, 15/22), with *NTRK3* (18.2%, 4/22) and *NTRK2* (13.6%, 3/22) comprising subsets of cases. Overall, there were 29 unique fusion partners. Several rearrangements were recurrent, including *BCAN-NTRK1* (*n* = 4), *TPM3-NTRK1* (n = 4), *ETV6-NTRK3* (n = 4), *ARHGEF2-NTRK1* (*n* = 2), *LMNA-NTRK1* (n = 2), *BCR-NTRK2* (n = 2), and *TRIM24-NTRK2* (n = 2). In our cohort, no fusion partner was shared by more than one *NTRK* gene. Furthermore, intrachromosomal rearrangements comprised the vast majority of *NTRK1* fusions (95.0%, 19/20), whereas interchromosomal rearrangements comprised most fusions involving *NTRK2* (85.7%, 12/14) and *NTRK3* (75.0%, 6/8). With the available methods, we were confident that *NTRK* was the 3′ fusion partner in 40 out of 42 tumors. (Table 2) [24, 32].

**Table 2:** *NTRK* rearrangements

Case	Assay detecting NTRK fusion	NTRK fusion	5’ fusion partner breakpoint or transcript	3’ fusion partner breakpoint or transcript
23	DNA-based NGS	ARGLU1-NTRK1	Exon 3	Exon 11
29	DNA-based NGS	ARHGEF11-NTRK1	Exon 39	Exon 10
10	RNA-based fusion panel	ARHGEF2-NTRK1	Exon 21	Exon 10
40	DNA-based NGS	ARHGEF2-NTRK1	Exon 21	Exon 9
27	DNA-based NGS	BCAN-NTRK1	Exon 13	Exon 8
30	DNA-based NGS	BCAN-NTRK1	Exon 6	Exon 8
31	DNA-based NGS	BCAN-NTRK1	Exon 14	Exon 11
32	DNA-based NGS	BCAN-NTRK1	Exon 13	Exon 9
8	DNA-based NGS	CD247-NTRK1	Intron 1	Exon 15
42	DNA-based NGS	CHTOP-NTRK1	Exon 5	Exon 8
21	RNA-based fusion panel	KIF21B-NTRK1^a^	Unknown	Unknown
9	DNA-based NGS	LMNA-NTRK1	Intron 5	Intron 10
41	DNA-based NGS	LMNA-NTRK1	Exon 12	Exon 11
26	DNA-based NGS	NTRK1-NFASC	Exon 7	Exon 3
24	DNA-based NGS	NOS1AP-NTRK1	Exon 10	Exon 9
33	DNA-based NGS	TPM3-NTRK1	Exon 10	Exon 12
36	DNA-based NGS	TPM3-NTRK1	Exon 7	Exon 12
37	DNA-based NGS	TPM3-NTRK1	Exon 10	Exon 9
38	DNA-based NGS	TPM3-NTRK1	Exon 7	Exon 8
19	RNA-based fusion panel	TPR and NTRK1^a,b^	Unknown	Unknown
25	DNA-based NGS	AFAP1-NTRK2	Exon 14	Exon 12
11	DNA-based NGS	BCR-NTRK2	Intron 1	Intron 12
28	DNA-based NGS	BCR-NTRK2	Exon 1	Exon 13
5	RNA-based fusion panel	EML1-NTRK2	Exon 2	Exon 16
4	RNA-based fusion panel	GKAP1-NTRK2	Exon 9	Exon 15
15	RNA-based fusion panel	KANK1-NTRK2	Exon 12	Exon 3
16	RNA-based fusion panel	KCTD16-NTRK2	Exon 3	Exon 16
1	RNA-based fusion panel	NBPF20-NTRK2	Exon 16	Exon 15
39	DNA-based NGS	PAIP1-NTRK2	Exon 9	Exon 13
18	RNA-based fusion panel	QKI-NTRK2	Exon 6	Exon 16
2	RNA-based fusion panel	SPECC1L-NTRK2	Exon 11	Exon 17
22	RNA-based NGS	TNS3-NTRK2^a^	Exon 16	Exon 12
3	RNA-based fusion panel	TRIM24-NTRK2	Exon 12	Exon 15
17	RNA-based fusion panel	TRIM24-NTRK2^c^	Exon 12	Exon 15, exon 16
34	DNA-based NGS	DLG1-NTRK3	Exon 10	Exon 11
6	RNA-based fusion panel	ETV6-NTRK3	Exon 4	Exon 14
14	RNA-based fusion panel	ETV6-NTRK3	Exon 4	Exon 14
20	DNA-based NGS	ETV6-NTRK3	Intron 4	Intron 12
35	DNA-based NGS	ETV6-NTRK3	Exon 5	Exon 15
12	RNA-based fusion panel	FRY-NTRK3	Exon 1	Exon 14
13	DNA-based NGS	KIAA1199-NTRK3	Intron 1	Intron 5
7	DNA-based NGS	MYO5A-NTRK3	Intron 33	Exon 9

^a^*NTRK* rearrangement confirmed by fluorescence in situ hybridization (FISH). ^b^ Unknown if *NTRK* is 5′ or 3′ fusion partner. ^c^*T*wo *NTRK2* fusions detected, with the major form fused to exon 16 of *NTRK2* and the minor form fused to exon 15 of *NTRK2*. *NGS* Next generation sequencing

### Histology of *NTRK*-fused gliomas

**Fig. 4:**
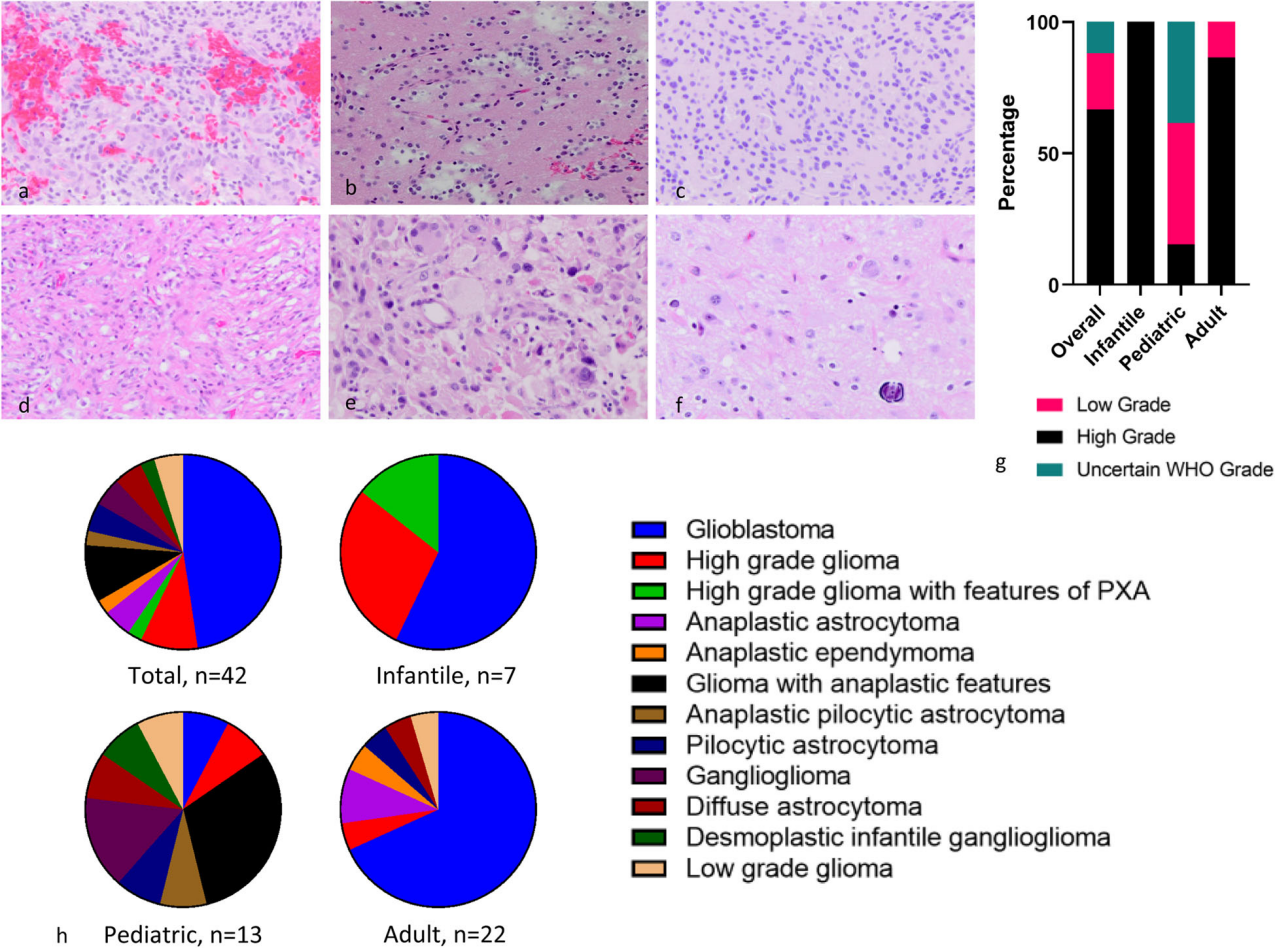
(Top-left) The histologic spectrum of *NTRK*-fused gliomas can include (**a**) glioblastoma (GBM), (**b**) infiltrative low-grade glioma, (**c**) glioma with anaplastic features and uncertain WHO grade, (**d**) pilocytic astrocytoma, (**e**) pleomorphic xanthoastrocytoma, and (**f**) ganglioglioma. (Top right) Histologic grades of *NTRK*-fused gliomas: most *NTRK*-fused gliomas demonstrate high-grade histology, particularly in tumors diagnosed in infant and adult patients. In contrast, the vast majority of *NTRK*-fused gliomas diagnosed in pediatric patients are of low-grade histology or of uncertain WHO grade. (Bottom) Histologic diagnoses of *NTRK*-fused gliomas: there were 12 unique histologic diagnoses assigned to *NTRK*-fused glioma in the study. Slightly less than half of all cases were diagnosed as GBM. Within the infantile and adult age cohorts, the majority of cases were diagnosed as GBM. A more diverse spectrum of tumors was diagnosed in the pediatric age cohort for which there was no single predominant histologic diagnosis

The histologic grade and diagnosis of *NTRK*-fused gliomas were heterogeneous. All infantile cases were histologically high-grade (100%, 7/7), including one with features of pleomorphic xanthoastrocytoma (PXA). Most adult cases were also histologically high-grade (86.4%, 19/22), with the majority being diagnosed as GBM (68.2%, 15/22). Interestingly, the adult cohort included a tumor with morphology and immunohistochemical profile indistinguishable from anaplastic ependymoma, but the molecular test results were more in keeping with a glioblastoma. The pediatric cohort was enriched in cases with low (46.2%, 6/13) or uncertain WHO grade (38.5%, 5/13), with fewer cases demonstrating high-grade histology (15.4%, 2/13). This was the group with the most histologic diversity, and the diagnoses included ganglioglioma (GG), diffuse astrocytoma (DA), glioblastoma (GBM), anaplastic pilocytic astrocytoma (APA), and desmoplastic infantile ganglioglioma (DIGG). The distribution of histologic grade and diagnosis by age group, as well as representative photos illustrating various histologic diagnoses are included in Fig. 4a-f. The distribution of *NTRK*-fused gliomas by histologic grade (low, high, and uncertain WHO grade) is summarized in Fig. 4g and the distribution of *NTRK*-fused gliomas by histologic diagnosis is summarized in Fig. 4h. The histologic diagnosis of each case is also listed in Table 3.

**Table 3:** Histologic diagnosis and methylation class (most with low confidence score)

Case	Age (years)	Histologic diagnosis	NTRK fusion	Match to known methylation family?	Closest methylation family match	Methylation family score
1	24	Low grade glioma	NBPF20-NTRK2	No	LGG, DNT	0.6959
2	16	Glioma with anaplastic features	SPECC1L-NTRK2	No	DMG, K27	0.4858
3	0.42	High grade glioma with features of pleomorphic xanthoastrocytoma	TRIM24-NTRK2	**Yes**	PXA	0.989
4	6	Low grade glioma	GKAP1-NTRK2	No	MTGF_PA	0.3584
5	0.23	High grade glioma	EML1-NTRK2	No	MTGF_PLEX_T	0.4474
6	4	Glioma with anaplastic features	ETV6-NTRK3	No	MTGF_PLEX_T	0.5473
7	42	Anaplastic ependymoma, WHO grade 3	MYO5A-NTRK3	No	MTGF_PLEX_T	0.19
8	42	Glioblastoma, WHO grade 4	CD247-NTRK1	N/A	N/A	N/A
9	43	Glioblastoma, WHO grade 4	LMNA-NTRK1	No	MTGF_GBM	0.6923
10	31	High grade glioma	ARHGEF2-NTRK1	No	DLGNT	0.2434
11	27	Glioblastoma, WHO grade 4	BCR-NTRK2	N/A	N/A	N/A
12	38	Anaplastic astrocytoma, WHO grade 3	FRY-NTRK3	No	MTGF_PLEX_T	0.4062
13	54	Glioblastoma, WHO grade 4	KIAA1199-NTRK3	N/A	N/A	N/A
14	0.25	Glioblastoma, WHO grade 4	ETV6-NTRK3	**Yes**	IHG	0.9836
15	2.7	Anaplastic pilocytic astrocytoma	KANK1-NTRK2	No	PXA	0.7403
16	7	Ganglioglioma, WHO grade 1	KCTD16-NTRK2	No	MTGF_PA	0.2699
17	9	Ganglioglioma, WHO grade 1	TRIM24-NTRK2	No	MTGF_PA	0.6814
18	16	Diffuse astrocytoma, WHO grade 2	QKI-NTRK2	N/A	N/A	N/A
19	2	Glioma with anaplastic features	TPR and NTRK1	No	MTGF_PA	0.3765
20	<1	Glioblastoma, WHO grade 4	ETV6-NTRK3	No	MTGF_PLEX_T	0.1241
21	1.83	High grade glioma	KIF21B-NTRK1	No	MTGF_GBM	0.773
22	7	Glioma with anaplastic features	TNS3-NTRK2	No	DLGNT	0.6739
23	55	Anaplastic astrocytoma, WHO grade 3	ARGLU1-NTRK1	N/A	N/A	N/A
24	<1	Glioblastoma, WHO grade 4	NOS1AP-NTRK1	N/A	N/A	N/A
25	16	Pilocytic astrocytoma, WHO grade 1	AFAP1-NTRK2	N/A	N/A	N/A
26	26	Diffuse astrocytoma, WHO grade 2	NTRK1-NFASC	N/A	N/A	N/A
27	48	Glioblastoma, WHO grade 4	BCAN-NTRK1	N/A	N/A	N/A
28	8	Glioblastoma, WHO grade 4	BCR-NTRK2	N/A	N/A	N/A
29	79	Glioblastoma, WHO grade 4	ARHGEF11-NTRK1	N/A	N/A	N/A
30	59	Glioblastoma, WHO grade 4	BCAN-NTRK1	N/A	N/A	N/A
31	54	Glioblastoma, WHO grade 4	BCAN-NTRK1	N/A	N/A	N/A
32	24	Pilocytic astrocytoma, WHO grade 1	BCAN-NTRK1	N/A	N/A	N/A
33	3	Desmoplastic infantile ganglioglioma, WHO grade 1	TPM3-NTRK1	N/A	N/A	N/A
34	52	Glioblastoma, WHO grade 4	DLG1-NTRK3	N/A	N/A	N/A
35	1	High grade glioma	ETV6-NTRK3	N/A	N/A	N/A
36	<1	Glioblastoma, WHO grade 4	TPM3-NTRK1	N/A	N/A	N/A
37	63	Glioblastoma, WHO grade 4	TPM3-NTRK1	N/A	N/A	N/A
38	78	Glioblastoma, WHO grade 4	TPM3-NTRK1	N/A	N/A	N/A
39	45	Glioblastoma, WHO grade 4	PAIP1-NTRK2	N/A	N/A	N/A
40	31	Glioblastoma, WHO grade 4	ARHGEF2-NTRK1	N/A	N/A	N/A
41	81	Glioblastoma, WHO grade 4	LMNA-NTRK1	N/A	N/A	N/A
42	44	Glioblastoma, WHO grade 4	CHTOP-NTRK1	N/A	N/A	N/A

*N/A* Not applicable, *LGG, DNT* Low-grade glioma, dysembryoplastic neuroepithelial tumor, *DMG, K27* Diffuse midline glioma H3 K27M mutant, *PXA* Pleomorphic xanthoastrocytoma, *MTGF_PA* Methylation group family_pilocytic astrocytoma, *MTGF_PLEX_T* Methylation group family_plexus tumor, *MTGF_GBM* Methylation group family_glioblastoma IDH wildtype, *DLGNT* Diffuse leptomeningeal glioneuronal tumor, *IHG* Infantile hemispheric glioma.

### Molecular features of *NTRK*-fused gliomas

**Fig. 5:**
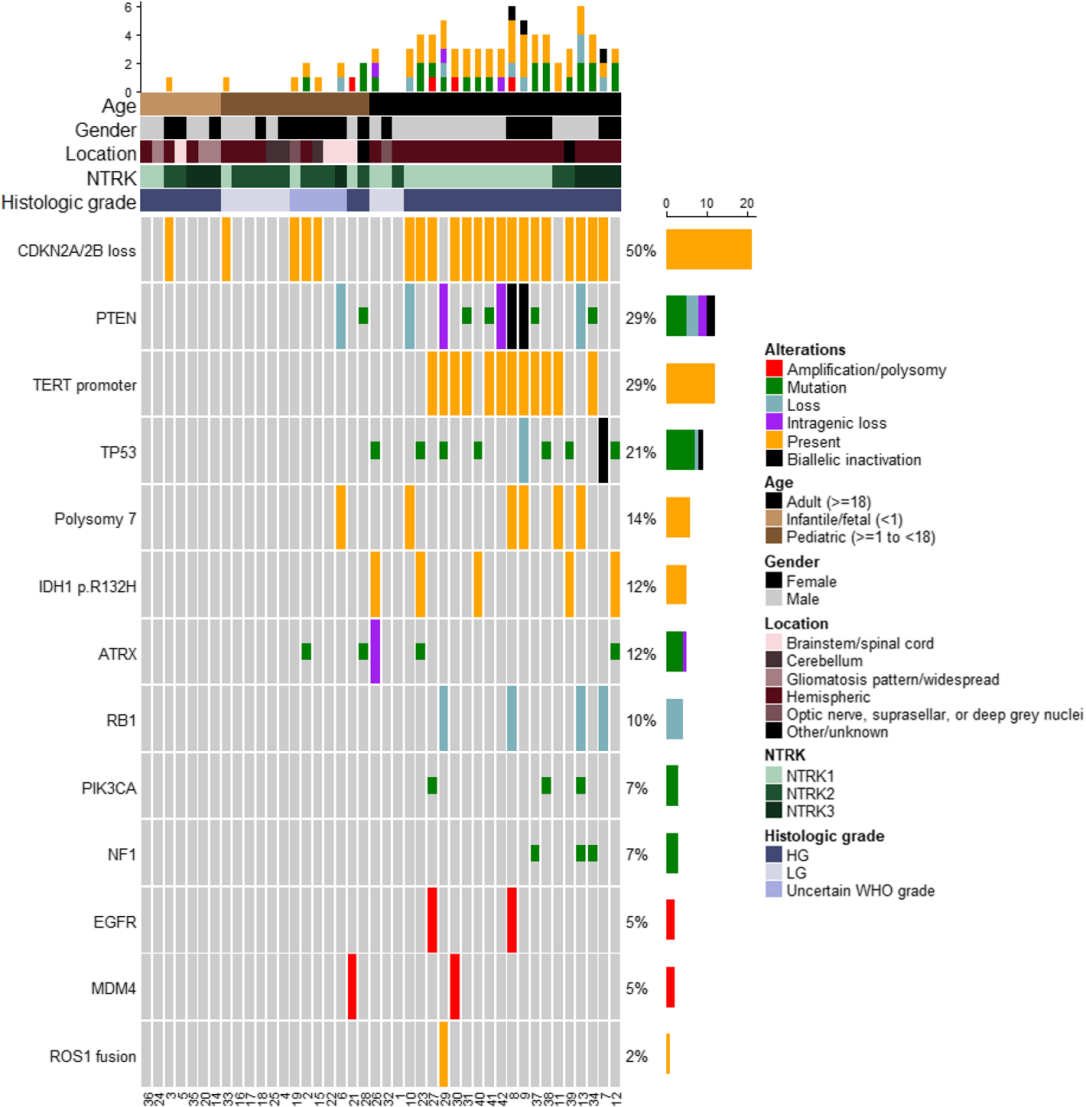
Oncoprint detailing common molecular alterations in *NTRK*-fused gliomas and patient clinical characteristics

*NTRK*-fused gliomas in our cohort demonstrated concurrent aberrations involving *CDKN2A/2B*, *TERT* promoter, *TP53*, *PTEN*, *EGFR*, *ATRX*, *RB1*, *IDH1*, polysomy 7, *ROS1*, *PIK3CA*, *NF1*, and *MDM4*. The frequency of these genetic aberrations in *NTRK*-fused gliomas increased with patient age cohort and seemed to correlate with histological grade in the pediatric and adult cohorts. Within the infantile cohort, there were no significant mutations and copy number changes beyond the *NTRK* fusion; the exception was case 3, a high-grade glioma with histology most consistent with anaplastic PXA that showed *CDKN2A/2B* loss. Within the pediatric cohort, detected aberrations included *CDKN2A/2B* loss (30.8%, 4/13), *ATRX* mutation (15.4%, 2/13), *PTEN* loss/mutation (15.4%, 2/13), polysomy 7 (7.7%, 1/13), and *MDM4* amplification (7.7%, 1/13); these were restricted to cases with high-grade histology or concerning histology of uncertain WHO grade; the only exception was case 33, diagnosed as DIGG, that had *CDKN2A/B* homozygous deletion. Within the adult cohort, detected aberrations included *CDKN2A/2B* loss (72.7%, 16/22), *TERT* promoter mutation (54.5%, 12/22), *PTEN* mutation/biallelic inactivation/(intragenic) loss (45.5%, 10/22), *TP53* mutation/ biallelic inactivation/loss (40.9%, 9/22), *IDH1* p.R132H mutation (22.7%, 5/22), polysomy 7 (22.7%, 5/22), *RB1* loss (18.2%, 4/22), *ATRX* mutation/intragenic loss (13.6%, 3/22), *PIK3CA* mutation (13.6%, 3/22), *EGFR* amplification (9.1%, 2/22), and *MDM4* amplification (4.5%, 1/22). The *IDH*-mutated gliomas were all negative for 1p/19q co-deletion, in keeping with diffuse astrocytomas. An oncoprint containing the major co-occurring genetic alterations along with clinicopathologic characteristics and tumor histology is provided in Fig. 5. Supplemental Table 2 provides all the genes with molecular alterations and chromosomal copy number changes for each case.

### Methylation profiling of *NTRK*-fused gliomas

**Fig. 6:**
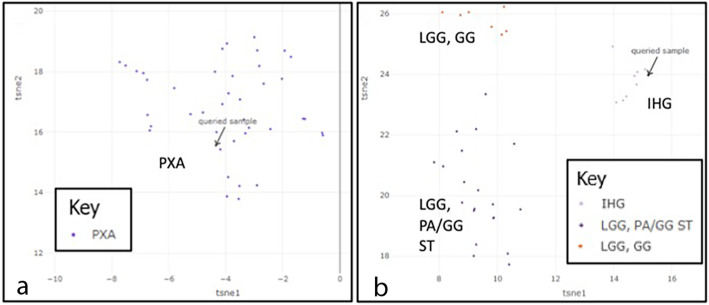
Methylation clustering analysis t-distributed stochastic neighbor embedding (tSNE) plots: of the 18 *NTRK*-fused gliomas with methylation profiling data, only two matched with high confidence to known methylation class families. (**a**) Case 3 (histologic diagnosis: high-grade glioma with features of pleomorphic xanthoastrocytoma (PXA)) matched to methylation class PXA, and (**b**) case 14 (histologic diagnosis: glioblastoma) matched to methylation class infantile hemispheric glioma (IHG). LGG, GG, low grade glioma, ganglioglioma; LGG, PA/GG ST, low grade glioma, pilocytic astrocytoma ganglioglioma spectrum in supratentorial compartment

Methylation profiling with clustering analysis was performed on 18 cases with available material. Two tumors matched to known methylation class families with high confidence (Fig. 6, Table 3): case 3, an infantile HGG with features of PXA, matched to methylation class family PXA (calibrated score = 0.989) and case 14, a 3-month-old with a histologic diagnosis of GBM, matched to infantile hemispheric glioma (IHG, calibrated score = 0.9836). In both instances, the histology was consistent with the matched methylation class family. All other cases matched with low confidence or not at all to known methylation class families (i.e. scores were lower than the recommended threshold value of ≥0.9 [7] or the less conservative threshold of ≥0.84 [8]. Seven cases had methylation classifier scores between 0.5 and 0.84 [8], with 2 (cases 9 and 21) having histology consistent with the closest methylation class family. Overall, a disproportionately high number of case either classified with calibrated score < 0.9 or did not classify with any reference cohort compared to previously published data [7] suggesting perhaps that *NTRK* fusions alter the DNA methylation pattern from non-*NTRK* driven cases of similar histology (Table 3; also please see Supplemental Table 3 for link to all methylation reports and t-sne plots).

**Fig. 7:**
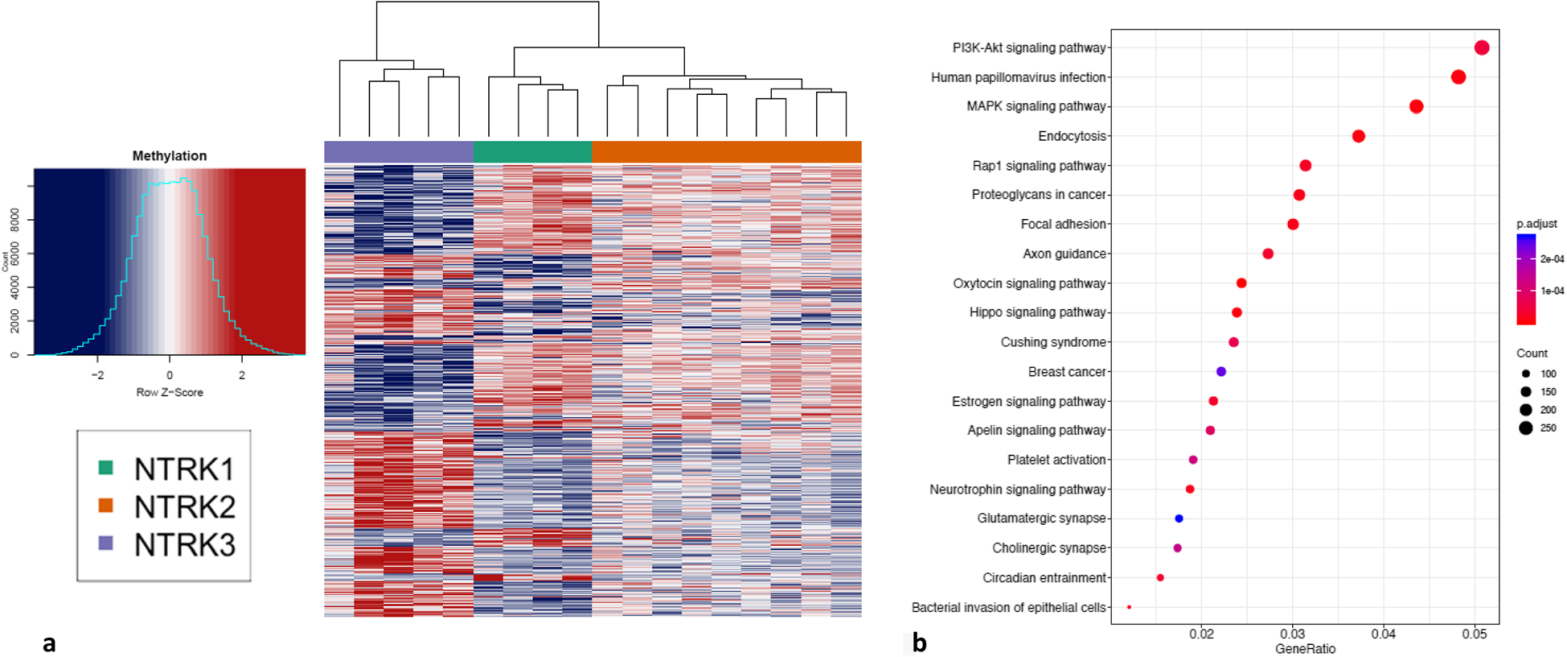
(**a**) Heatmap of the top 10,000 differentially methylated genes/probes by *NTRK* gene involved (blue indicates hypomethylation, and red indicates hypermethylation). (**b**) KEGG pathway analysis reveals several pathways enriched in the top differentially methylated gene/probes. The dot plots represent the ratio of genes (x-axis) involved in each signaling pathway (y-axis). The size of the dots shows the gene counts, and the color denotes the significance level

While some newly described CNS tumor entities driven by gene fusions form unique entities [45], unsupervised PCA of the methylation profiles of *NTRK*-fused cohort showed no obvious grouping by *NTRK* gene involved, histologic grade, or age (Supplemental Fig. 1). KEGG pathway analysis of the top 10,000 most variably methylated genes/probes in the cohort demonstrated enrichment in pathways involving PI3K-AKT signaling (and related human papillomavirus infection signaling) and MAPK signaling, among others (Fig. 7).

## Discussion

With the recent FDA approval of larotrectinib, a selective pan-TRK inhibitor, and entrectinib, a selective pan-TRK, ROS1, and ALK inhibitor [42], there has been much interest in characterizing and diagnosing tumors with *NTRK* fusions. Both therapies have demonstrated significant treatment responses in *NTRK*-fused tumors [4, 13, 15–17, 23, 30], including CNS metastases [13, 16, 17, 23] and primary CNS tumors [1, 9, 16, 50, 57], and are generally well tolerated [4, 13, 15–17, 23, 30]. Our study addresses gaps in the knowledge of the clinical and molecular features of *NTRK*-fused gliomas. In addition, the study serves to corroborate features of *NTRK*-fused gliomas that have been previously described.

One of the findings of this multi-institutional study is the considerable clinicopathologic and molecular heterogeneity of *NTRK*-fused gliomas. *NTRK* fusions do not appear to define *ipso facto* a single glial entity but rather are a genetic feature occurring in multiple tumor types.

Clinically, we found that *NTRK*-fused gliomas can involve all CNS compartments but are primarily hemispheric in adults (90.9%) and infants (85.7%); the anatomic distribution of pediatric *NTRK*-fused gliomas is less predictable. During a median follow-up period of 23 months after diagnosis, 28.6% of patients died and 58.0% of patients showed evidence of recurrence/progression, events that were mostly associated with tumors with high-grade histology. A prior study showed a 5 year overall survival of 42.9% in young patients with hemispheric *NTRK*-fused gliomas [22]. However, *NTRK*-fused gliomas with low-grade histology may still exhibit an aggressive clinical course [26]. Ultimately, the prognosis of *NTRK*-fused gliomas may rapidly change with the more widespread use of targeted TRK inhibitors.

The diverse histology of *NTRK*-fused gliomas overlaps with entities such as DIGG, GG, PXA, PA (including anaplastic), DA grades 2 and 3, and GBM in our study. In keeping with the wide spectrum of *NTRK*-fused CNS tumor histology, *NTRK* rearrangements have been previously reported in GBM [9, 18, 19, 21, 28, 34, 38, 39, 41, 43, 44, 52, 53, 56], gliosarcoma [21], AA [9, 18, 21, 52], diffuse midline glioma / DIPG [9, 52], HGG [9, 22, 34, 57], glioneuronal tumor (including high grade) [1, 16, 29], pilocytic astrocytoma (PA) (including anaplastic) [9, 18, 21, 25, 35], low grade astrocytroma with features of PA [26], PXA [55], GG [1, 9, 36, 37], DIGG [6, 9], LGG [18, 34, 44, 50, 53], glioma, not otherwise specified [9, 18], neuroepithelial neoplasm [43], CNS fibroblastic tumor [47], primitive neuroectodermal tumor (PNET) [12], CNS embryonal tumor [14], and tumors with oligodendroglial or oligoastrocytic-like histology [12, 21, 29, 37, 55].

The present study adds to the literature by demonstrating that the histology and histologic grade of *NTRK*-fused gliomas vary by patient age. *NTRK*-fused gliomas in all infantile and most adult patients were histologically high-grade, with the majority diagnosed as GBM. In contrast, pediatric *NTRK*-fused gliomas were more likely to be of low-grade (46.2%) or uncertain WHO grade (38.5%), and there was no single predominant histologic diagnosis in this cohort. These features of the pediatric *NTRK*-fused gliomas make their diagnosis and clinical management difficult.

Gliomas with *NTRK* fusions have been previously reported to possess co-occurring genetic alterations such as *IDH* [18, 21, 39, 56], *H3.3* K27M [52], *H3F3A* [9], *EGFR* amplification [21, 28, 56], *EGFRvIII* [21], *PTEN* [9, 21, 28], *CDKN2A/2B* deletion [9, 22, 28, 39, 52, 55, 57], *CDKN2C* deletion [28], *TP53* mutations/inactivation [21, 39, 52, 57], and *ATRX* [39], among others [21]. Our study matches many of these molecular findings and further demonstrates that the frequency of pathologically significant mutations in *NTRK*-fused gliomas appears to increase with patient age. In addition, *TERT* promoter mutations are observed only in histologically high-grade adult tumors, *PTEN* alterations are almost exclusively seen in histologically high-grade tumors, and *CDKN2A/2B loss* is rare in histologically low-grade tumors.

Notably, 22.7% of adult *NTRK*-fused gliomas in our cohort are *IDH1* p.R132H mutated, raising questions about the oncogenic driver event in these specific tumors and whether they display oncogenic dependence on the *NTRK* fusion. In one case report of secondary *IDH*-mutant GBM [39], an *NTRK* fusion was detected in only a subclonal tumor population and was absent in the original AA, suggesting that the *NTRK* fusion was a secondary alteration. In contrast, in *NTRK*-fused gliomas without co-occurring pathologically significant mutations [9, 21, 52], typically arising in younger patients, *NTRK* fusions are almost certainly the oncogenic driver event. This is supported by multiple in vivo models that have demonstrated the capability of *NTRK* fusions to drive gliomagenesis/tumorgenesis [11, 28, 33, 51, 52].

In our series, gliomas with rearrangements involving the same *NTRK* gene and fusion partner do not necessarily have the same histology or methylation class, which suggests that other factors such as age, co-occurring genetic events, cell of origin and microenvironment potentially play an important role in tumor biology. We have also observed that the *NTRK* gene involved in the rearrangement differs in frequency by age, with pediatric gliomas having a high percentage of rearrangements involving *NTRK2* (69.2%), and adult gliomas having a high percentage of rearrangements involving *NTRK1* (68.2%).

Most *NTRK*-fused gliomas in our cohort were not a perfect match with known methylation entities. Only two cases matched with high confidence to methylation class families. Furthermore, cases that matched with low confidence generally had histology that was not characteristic of the methylation class family, mirroring the experience described in a prior case study of *NTRK*-fused glioneuronal tumor [29]. Another study reported a proportion of *NTRK*-fused gliomas matching to methylation classes including IHG, diffuse midline glioma H3 K27M mutant, PXA, and GBM, IDH wildtype, subclass midline [9]. Overall, the findings from our study and prior studies with methylation data [9, 29] suggest that better methylation profile classifier guidelines are needed to account for *NTRK*-fused. Our unsupervised PCA demonstrating no obvious grouping by *NTRK* gene involved, age, or histology highlights the heterogeneity within the *NTRK*-fused glioma methylome.

In summary, *NTRK*-fused gliomas are clinically, histologically, and molecularly diverse, with notable differences by age group and associated genetic alterations. Additional studies are needed to develop clinical guidelines for the diagnostic workup of potential *NTRK*-fused CNS tumors and to improve methylation classifier guidelines for *NTRK*-fused gliomas. Further mechanistic work is required to determine the role of *NTRK* fusions in driving gliomagenesis in the setting of concurrent oncogenic drivers such as *IDH* mutations and to demonstrate how downstream TRK signaling pathways may be mediated by different *NTRK* gene involved, location of *NTRK* fusion breakpoint, fusion partner, and cell of origin.

## Supplementary information

**Additional file 1: Supplemental figure 1.** Unsupervised principal component analysis (PCA) of methylation profiles of *NTRK*-fused gliomas demonstrates that no homogenous groups form when correlated with (**a**) *NTRK* gene involved, (**b**) patient age, or (**c**) histologic grade.

**Additional file 2: Supplemental tables.** (**1**) methods for each case; (**2**) single nucleotide variants and copy number variants encountered in each case; (**3**) legend for the methylation reports and t-sne plots corresponding to each case.
