# Supplementary figures and images for: Molecular and clinicopathologic features of gliomas harboring NTRK fusions
Source: Acta Neuropathol Commun. 2020 Jul 14;8:107. doi: 10.1186/s40478-020-00980-z (PMC7362646; doi:10.1186/s40478-020-00980-z)

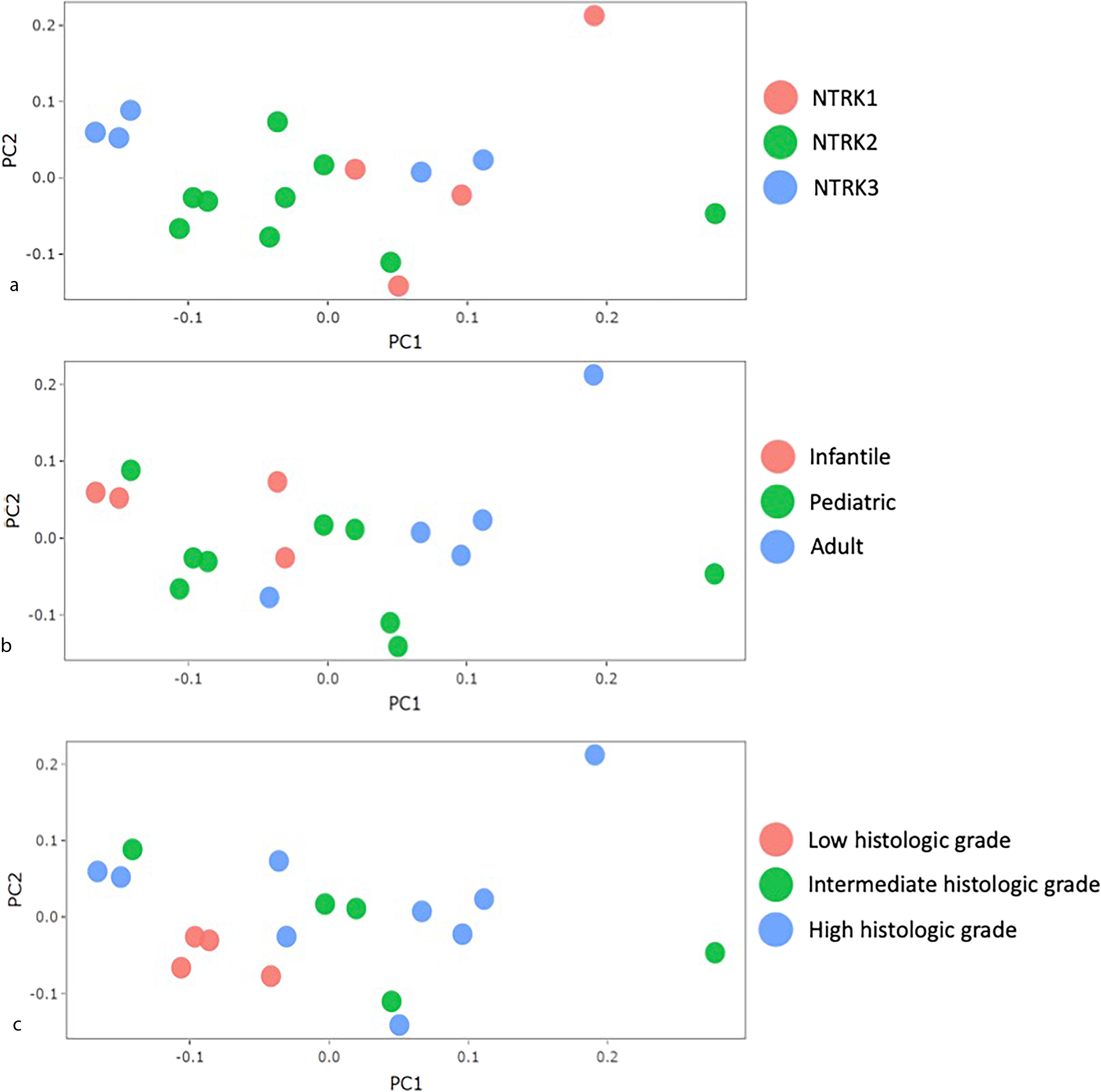

Supplement: Supplementary file 1 — Additional file 1: Supplemental figure 1. Unsupervised principal component analysis (PCA) of methylation profiles of NTRK-fused gliomas demonstrates that no homogenous groups form when correlated with (a) NTRK gene involved, (b) patient age, or (c) histologic grade. [file 40478_2020_980_MOESM1_ESM.tif]
