# Supplementary material for: Molecular and clinicopathologic features of gliomas harboring NTRK fusions
Source: Acta Neuropathol Commun. 2020 Jul 14;8:107. doi: 10.1186/s40478-020-00980-z (PMC7362646; doi:10.1186/s40478-020-00980-z)
Supplement: Supplementary file 2 — Additional file 2: Supplemental tables. (1) methods for each case; (2) single nucleotide variants and copy number variants encountered in each case; (3) legend for the methylation reports and t-sne plots corresponding to each case. [file 40478_2020_980_MOESM2_ESM.zip › Supplemental Table 3.docx]

|  | Methylation Report Number |
| --- | --- |
| 1 | RD-19-412 |
| 2 | RD-19-413 |
| 3 | RD-19-404 |
| 4 | RD-19-405 |
| 5 | RD-19-406 |
| 6 | RD-19-407 |
| 7 | RD-19-411 |
| 9 | RD-19-410 |
| 10 | RD-19-409 |
| 12 | RD-19-408 |
| 14 | RD-19-414 |
| 15 | RD-19-978 |
| 16 | RD-19-980 |
| 17 | RD-19-979 |
| 19 | RD-19-415 |
| 21 | RD-19-527 |
| 22 | RD-19-1027 |

Supplemental table 3 and link to methylation reports: [https://genome.med.nyu.edu/public/snuderllab/](https://urldefense.proofpoint.com/v2/url?u=https-3A__genome.med.nyu.edu_public_snuderllab_&d=DwMGaQ&c=qS4goWBT7poplM69zy_3xhKwEW14JZMSdioCoppxeFU&r=Meixn-dc57BavP3YEA8f5UGpRV-9bFipvCDdhpQeyqh_EppoKYwnWOt7B4QV4xPT&m=fi-Alg-vrdyr08G3nwu9_3EHUlaNwl347f_TNdZnCqc&s=mzKH6bY2WXCtbviEQNYMrIqng9S_Xu2L2sA__oOr-Uc&e=)
